# Adaptive Features of Natural Killer Cells in Multiple Sclerosis

**DOI:** 10.3389/fimmu.2019.02403

**Published:** 2019-10-15

**Authors:** Antía Moreira, Elisenda Alari-Pahissa, Elvira Munteis, Andrea Vera, Ana Zabalza, Mireia Llop, Noelia Villarrubia, Marcel Costa-García, Roberto Álvarez-Lafuente, Luisa María Villar, Miguel López-Botet, Jose E. Martínez-Rodríguez

**Affiliations:** ^1^Neurology Department, Hospital del Mar Medical Research Institute (IMIM), Barcelona, Spain; ^2^Neurology Department, Althaia, Xarxa Assistencial i Universitària de Manresa, Manresa, Spain; ^3^Departament de Medicina, Universitat Autònoma de Barcelona, Barcelona, Spain; ^4^University Pompeu Fabra, Barcelona, Spain; ^5^Immunology Department, Hospital Universitario Ramón y Cajal, Madrid, Spain; ^6^Neurology Service, Instituto de Investigación Sanitaria del Hospital Clínico de San Carlos, Madrid, Spain; ^7^Hospital del Mar Medical Research Institute (IMIM), Barcelona, Spain

**Keywords:** multiple sclerosis, natural killer cells, cytomegalovirus, NKG2C, FcεRIγ, PLZF

## Abstract

Human cytomegalovirus (HCMV) has been recently related with a lower susceptibility to multiple sclerosis (MS). HCMV promotes an adaptive development of NK cells bearing the CD94/NKG2C receptor with a characteristic phenotypic and functional profile. NK cells are proposed to play an immunoregulatory role in MS, and expansion of the NKG2C(+) subset was recently associated with reduced disability progression. To further explore this issue, additional adaptive NK cell markers, i.e., downregulation of FcεRIγ chain (FcRγ) and PLZF transcription factor, as well as antibody-dependent NK cell activation were assessed in controls and MS patients considering HCMV serology and clinical features. In line with previous reports, increased proportions of NKG2C(+), FcRγ(–), and PLZF(–) CD56^dim^ NK cells were found in HCMV(+) cases. However, PLZF(–) NK cells were detected uncoupled from other adaptive markers within the CD56^bright^ subset from HCMV(+) cases and among CD56^dim^ NK cells from HCMV(–) MS patients, suggesting an additional effect of HCMV-independent factors in PLZF downregulation. Interferon-β therapy was associated with lower proportions of FcRγ(–) CD56^dim^ NK cells in HCMV(+) and increased PLZF(–) CD56^bright^ NK cells in HCMV(–) patients, pointing out to an influence of the cytokine on the expression of adaptive NK cell-associated markers. In addition, proportions of NKG2C(+) and FcRγ(–) NK cells differed in progressive MS patients as compared to controls and other clinical forms. Remarkably, an adaptive NK cell phenotype did not directly correlate with enhanced antibody-triggered degranulation and TNFα production in MS in contrast to controls. Altogether, our results provide novel insights into the putative influence of HCMV and adaptive NK cells in MS.

## Introduction

Multiple sclerosis (MS) is an immune-mediated disease of the central nervous system characterized by a highly variable clinical course. Both genetic and environmental factors are involved in MS, with an influence of herpesvirus infections supported by seroepidemiological studies. Whereas Epstein–Barr virus (EBV) is consistently associated with MS and might play a pathogenic role as an environmental trigger (1, 2), human cytomegalovirus (HCMV) was recently related to lower MS susceptibility (3–6); however, the basis for these observations remains uncertain. HCMV establishes a lifelong latent infection whose prevalence appears inversely related with socioeconomical development and, in line with the “hygiene hypothesis,” the reported associations with MS might be indirectly linked to the exposure to other environmental factors. On the other hand, a putative influence of HCMV in MS immunopathology is conceivable (7), as this herpesvirus may exert a profound impact on the immune system (8), and could potentially modulate the response to other pathogens (i.e., heterologous immunity) (9, 10). Nevertheless, whether HCMV may exert a beneficial or detrimental effect on MS is currently uncertain (11).

T lymphocytes and natural killer (NK) cells play complementary key roles in controlling HCMV, which, reciprocally, has developed a variety of immune evasion mechanisms (12). HCMV has been reported to induce a persistent reconfiguration of NK cell compartment, both in healthy individuals and under pathological conditions. The magnitude of this effect is variable and hallmarked by the differentiation and expansion of functionally mature NK cells displaying high levels of the CD94/NKG2C activating receptor encompassed by additional phenotypic and functional changes (13–16). Human adaptive NKG2C(+) NK cells are contained within the major circulating CD56^dim^ subset, which mediates cytotoxicity and cytokine production upon interaction with target cells, either directly or through IgG engagement of FcγRIIIA (CD16A). As compared to other subsets, adaptive NKG2C(+) NK cells effectively mediate antibody-dependent effector functions, particularly cytokine production (17, 18). Additional associated-adaptive NK cell markers include epigenetic silencing of the promyelocytic leukemia zinc finger (PLZF) transcription factor, with unknown functional implications in NK cells (17, 19–21), and loss of the FcεRI gamma chain (FcRγ) adaptor coupled to CD16, whose replacement by the ζ chain may enhance antibody-dependent NK cell activation (17, 19, 22, 23). Some adaptive NK cell-associated features have also been detected in NKG2C(–) NK cell subsets, suggesting that NK cell differentiation is independent of NKG2C expression and may be induced by stimuli other than HCMV (17, 18, 24). Moreover, adaptive NK cell differentiation may not be a coordinated process, as supported by the delayed FcRγ loss observed in adaptive NK cells following HCMV infection in hematopoietic stem cell transplantation recipients (25). The cellular and molecular mechanisms underlying the development of adaptive NK cells and their implications in human health remain partially defined.

NK cells have been previously studied in the context of MS (26, 27), and changes associated with the response to some immunomodulatory therapies have been reported (28, 29), proposing an immunoregulatory role played by the minor circulating CD56^bright^ NK cell subset (30). These cells display a CD16(–) NKG2A(+) phenotype and have a limited cytotoxic capacity but efficiently produce cytokines, constituting the predominant subset in peripheral lymphoid tissues. However, the role of NK cells in MS immunology is only partially understood. Recently, we described an association of HCMV-induced expansion of NKG2C(+) NK cells with a lower risk of disability progression in MS, suggesting an influence of these lymphocytes on the clinical course (31). In the present study, we aimed to further explore whether HCMV and adaptive NK cells play a protective role in MS. Adaptive NK cell immunophenotype and antibody-triggered NK cell activation against EBV-transformed B cell lines were evaluated in MS patients considering their HCMV serostatus and clinical profile, providing further insights into the involvement of NK cells in this disease.

## Materials and Methods

### Study Design and Population

A case–control study of MS patients was designed to analyze adaptive NK cell markers and antibody-dependent NK cell activation in accordance to HCMV serostatus and clinical characteristics. MS patients diagnosed based on McDonald criteria 2010 from Hospital del Mar, IMIM (Barcelona) and Hospital Ramón y Cajal (Madrid) (REEM, Spanish Network for MS research) were recruited from routine clinical visits. In addition, a subgroup of MS patients was prospectively evaluated after onset of interferon-beta (IFNβ) therapy to assess the influence of this treatment on adaptive NK cells. We excluded patients on disease-modifying therapies known to deplete peripheral lymphocytes or alter their trafficking (e.g., natalizumab, fingolimod, dimethyl fumarate), as well as those undergoing corticosteroid treatment in the last 30 days, pregnancy, or any severe concomitant disease. The following demographic and clinical characteristics were evaluated in every case: age, sex, disease duration, MS form (RRMS: relapsing–remitting MS, SPMS: secondary progressive MS, PPMS: primary progressive MS), disability scores (EDSS: Expanded Disability Status Scale, MSSS: multiple sclerosis severity score) and treatment. Healthy controls matched for age and sex were recruited from the same geographical area.

The study was approved by the local Ethics Committee, including participants after written informed consent. Blood samples obtained from venous puncture were obtained from controls and MS patients, performing standard clinical diagnostics test to evaluate EBV-(LIASON®) and HCMV-specific circulating antibodies (Roche®, Cobas602).

### Analysis of NK Cell Immunophenotype

**Figure 1:**
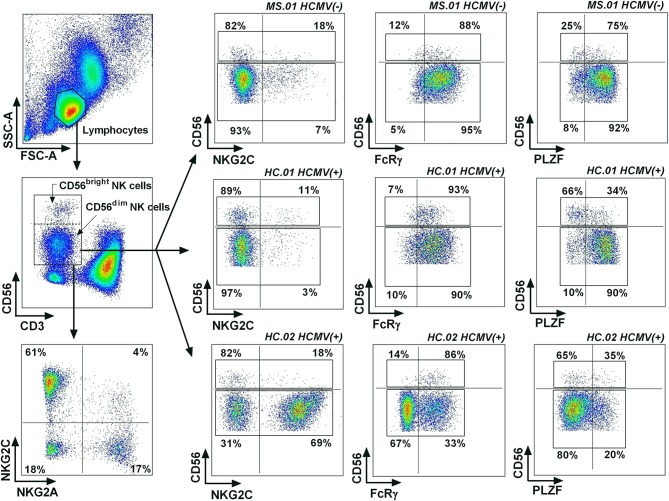
Gating strategy for adaptive NK cells. Lymphocytes were identified based on their forward scatter (FCS) and side scatter (SSC) characteristics, defining NK cells as CD3(–) CD56(+) lymphocytes. Representative examples were selected based on the expression of adaptive NK cell markers, showing a case with a low expression of the three adaptive markers (MS.01), a case with low NKG2C(+), FcRγ(–), and PLZF(–) expression in CD56^dim^ NK cells but with a higher proportions of PLZF(–) CD56^bright^ NK cells (HC.01), and a case with higher proportions of NKG2C(+), FcRγ(–), and PLZF(–) CD56^dim^ NK cells.

Peripheral blood mononuclear cells (PBMCs) from 47 healthy controls and 151 MS patients were isolated from blood samples collected in EDTA tubes using Ficoll–Hypaque density gradient centrifugation and cryopreserved in fetal calf serum with 10% dimethyl sulfoxide until analysis. Sample staining for flow cytometry analysis was performed using anti-NKG2A (clone Z199) indirectly labeled with a secondary goat anti-mouse PE-Cy7 (Biolegend) and the following fluorochrome-conjugated antibodies: anti-CD3-PerCP (BD Pharmigen), anti-CD56-APC (Biolegend), antiCD16-eFluor450 (eBioscience), and NKG2C-PE (R&D Systems). For the analysis of FcRγ (MS, *n* = 139; controls *n* = 47) and PLZF expression (MS, *n* = 86; controls *n* = 26), cells were treated with a fixation/permeabilization kit (BD Biosciences) followed by incubation with anti-FcRγ-FITC (Millipore) and anti-PLZF-PE CF594 (BD Biosciences). Samples were acquired in LSRFortessa (BD Biosciences) and data were analyzed using FlowJo software (Tree Star, Oregon, USA), using the gating strategy shown in Figure 1.

### Functional Assessment of Antibody-Dependent NK Cell Activation

PBMCs from 42 MS patients (22 RRMS, 8 SPMS and 12 PPMS) and 17 controls matched for HCMV serostatus were incubated overnight at 37°C with recombinant IL-2 (200 U/ml). The response of NK cells to the HLA class I-defective 721.221 B-lymphoblastoid cell line with or without rituximab (50 ng/ml) was assessed following a 4-h incubation (E/T = 1/1). A complementary approach was performed using EBV(+) AKBM cells as targets following induction of the lytic cycle in the presence of EBV(+) or EBV(–) sera, as previously described (32, 33). Surface expression of CD107 as a marker of degranulation and intracellular TNFα production was analyzed by flow cytometry as previously reported (34), using the anti-CD107-APC (BD Pharmigen) monoclonal antibody during incubation together with monensin (GolgiStop® BD) and brefeldin (GolgiPlug® BD). Cultures were then stained with anti-CD3-PerCP (BD Pharmigen), anti-CD56-APC-Cy7 (Biolegend), and anti-NKG2C-PE (R&D System), permeabilized, fixed and stained intracellularly with anti-TNFα-CFBlue (labeled by Immunostep), anti-FcRγ-FITC (Millipore), and anti-PLZF-PE CF594 (BD Biosciences). Data acquisition was performed with an LSRFortessa cytometer (BD Biosciences).

### Multidimensional Flow Cytometry Analysis Using Barnes-Hut t-SNE

A multidimensional flow cytometry analysis was performed as previously described (35), compensating raw flow cytometry data using FlowJo software (Tree Star, Oregon, USA) and later imported into R using flowCore and openCyto packages. Lymphocytes were gated on forward scatter and side scatter characteristics and then on CD56^dim^ NK cells. FITC channel was normalized using flowStats R package in order to reduce experimental variability on fluorescence intensity. Subsequently, randomly selected data from 500 CD56^dim^ NK cells per sample was concatenated. The most positive and negative one per mille values for each parameter were reduced to their less extreme border. Next, Barnes-Hut t-SNE was conducted using the Rtsne package. Graphics were produced using ggplot2 and RcolorBrewer R packages.

### Statistical Analysis

Normal distribution was assessed using Kolmogorov–Smirnov test. Continuous variables were expressed as mean ± standard deviation (SD) or median (first–third quartile) for parametric and non-parametric variables, respectively. Relationship between continuous and dichotomous variables was assessed by Student's *t*-test or Mann–Whitney *U*-test, respectively. Pearson or Spearman correlation coefficients were calculated for pairwise comparison of continuous variables. Wilcoxon test for paired samples was used to compare matched prospectively followed-up samples before and after IFNβ therapy. Three-dimensional Venn analysis evaluated the co-expression of adaptive markers in NK cells. Multivariate lineal regression analysis determined predictors for adaptive NK cell markers adjusting the models for HCMV serostatus, clinical variables, and IFNβ treatment. Results were considered significant at the two-sided level of 0.05. Analyses were performed using SPSS v.23 software.

## Results

### HCMV-Related Expansion of Adaptive NK Cells in MS Patients

We comparatively studied in controls and MS patients the influence of HCMV on the expression of adaptive NK cell differentiation markers (NKG2C, FcRγ, and PLZF) based on the gating strategy for flow cytometry analysis illustrated in Figure 1. Demographic and clinical characteristics of MS patients and controls are shown in Table 1. In our study, HCMV seroprevalence was comparable in both groups (Table 1), and no clinical differences were observed in MS patients associated with HCMV serostatus (data not shown).

**Table 1:** Demographic and clinical characteristics of MS patients and controls.

	**Controls**	**MS patients**	*P*-value^*^	**RRMS**	**SPMS**	**PPMS**	***P*-value^#^**
	***n* = 47**	***n* = 151**		***n* = 88**	***n* = 44**	***n* = 19**	
Age (years)	46.6 ± 13.3	50.1 ± 11.4	0.08	45.6 ± 10.0	55.0 ± 8.8	59.4 ± 13.0	<0.001
HCMV seroprevalence, *n* (%)	37 (78.7)	103 (68.7)	0.126	59 (67.8)	33 (75)	11 (57.9)	0.217
EBV seroprevalence, *n* (%)	40 (87)	147 (98.7)	<0.01	86 (98.9)	43 (97.7)	18 (100)	0.152
Sex (female), *n* (%)	30 (63.8)	101 (66.9)	0.413	59 (67.0)	28 (63.6)	14 (73.7)	0.861
MS duration (years)	–	15.1 ± 10.0		11.6 ± 9.1	21.5 ± 8.7	16.2 ± 9.8	<0.001
DMT, *n* (%)	–	50 (33.1)		42 (47.7)	7 (15.9)	1 (5.3)	<0.001
DMT-naive, *n* (%)	–	47 (34.1)		22 (28.9)	9 (20.9)	16 (84.2)	<0.001
EDSS	–	3.5 (2.0–6.0)		2.0 (1.0–3.0)	6.5 (5.5–7.5)	6.0 (4.5–7.0)	<0.001
MSSS	–	4.23 ± 2.92		2.60 ± 2.20	6.58 ± 2.25	6.56 ± 1.91	<0.001
2y-RR	–	0.19 ± 0.41		0.34 ± 0.52	0.01 ± 0.08	0.0 ± 0.0	<0.001
ARR	–	0.37 ± 0.41		0.42 ± 0.42	0.52 ± 0.39	0.01 ± 0.02	<0.001

*Values are expressed as mean ± SD or median (1st−3rd quartile). HCMV, human cytomegalovirus; EBV, Epstein–Barr virus; DMT, disease-modifying therapy (interferon-beta, n = 45; glatiramer acetate, n = 5), EDSS, Expanded Disability Status Scale; MSSS, multiple sclerosis severity score; 2y-RR, relapsing rate in the previous 2 years; ARR, annualized relapsing rate; RRMS, relapsing–remitting MS; SPMS, secondary progressive MS; PPMS, primary progressive MS*.

* *P-values comparing controls and MS patients using t test or Mann–Whitney U-test*.

# *P-values comparing RRMS, SPMS, and PPMS patients using one-way ANOVA*.

**Figure 2:**
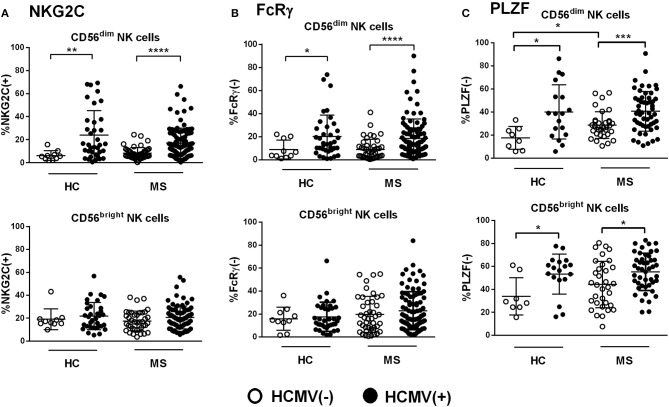
Expression of adaptive markers in NK cells from controls and MS patients according to HCMV serostatus. **(A)** Proportions of NKG2C(+), **(B)** FcRγ(–), and **(C)** PLZF(–) in CD56^dim^ and CD56^bright^ NK cells from healthy controls (HC) and MS patients according to HCMV serostatus. Higher proportions of NKG2C(+), FcRγ(–), and PLZF(–) in CD56^dim^ NK cells were found in HCMV(+) as compared to HCMV(–) cases **(A–C)**, in addition to higher proportion of PLZF(–) NK cells perceived in the CD56^bright^ subset **(C)**. Increased proportions of PLZF(–) CD56^dim^ NK cells were also found in HCMV(–) MS patients as compared to HCMV(–) controls **(C)**. NKG2C, MS = 151, HC 139; FcRγ, MS = 139, HC = 47; PLZF, MS = 86, PLZF = 26. Individuals with an *NKG2C* ^del/del^ genotype (HC, *n* = 1; MS, *n* = 6) were excluded from the analysis of NKG2C expression. *P* values (Mann–Whitney *U* test): * <0.05, ** <0.01, *** <0.001, **** <0.0001.

HCMV seropositivity was associated with greater proportions of NKG2C(+) NK cells, with no significant differences between MS patients and controls (Figure 2A). The expansion of NKG2C(+) cells was confined to the CD56^dim^ NK cell subset, whereas detection of CD56^bright^ NKG2C(+) NK cells appeared unrelated to HCMV serology (Figure 2A). Similarly, the frequency of CD56^dim^ FcRγ(–) NK cells was also increased in HCMV(+) patients and controls as compared to HCMV(–) individuals (Figure 2B). By contrast, HCMV(+) controls and MS patients showed increased proportions of PLZF(–) cells not only in CD56^dim^ NK cells but, remarkably, also among the CD56^bright^ subset (Figure 2C); these PLZF(–) NK cells were confirmed to display the characteristic CD56^bright^ NKG2A(+) CD16(–) phenotype (Supplementary Figure 1). Of note, an increased frequency of PLZF(–) cells was also detected among CD56^dim^ NK cells from HCMV(–) MS patients as compared to seronegative controls (Figure 2C). Overall, these results support previous studies describing the HCMV-related expansion of adaptive NK cells displaying the NKG2C(+), FcRγ(–), and PLZF(–) markers in CD56^dim^ NK cells; however, a lack of PLZF expression associated to HCMV seropositivity was also perceived in the CD56^bright^ NK cell subset, in both controls and MS patients, in addition to an influence of HCMV-independent factors in PLZF downregulation in CD56^dim^ NK cells found in MS cases.

### Relationship Between Adaptive NK Cell Markers in MS Patients and Controls

**Figure 3:**
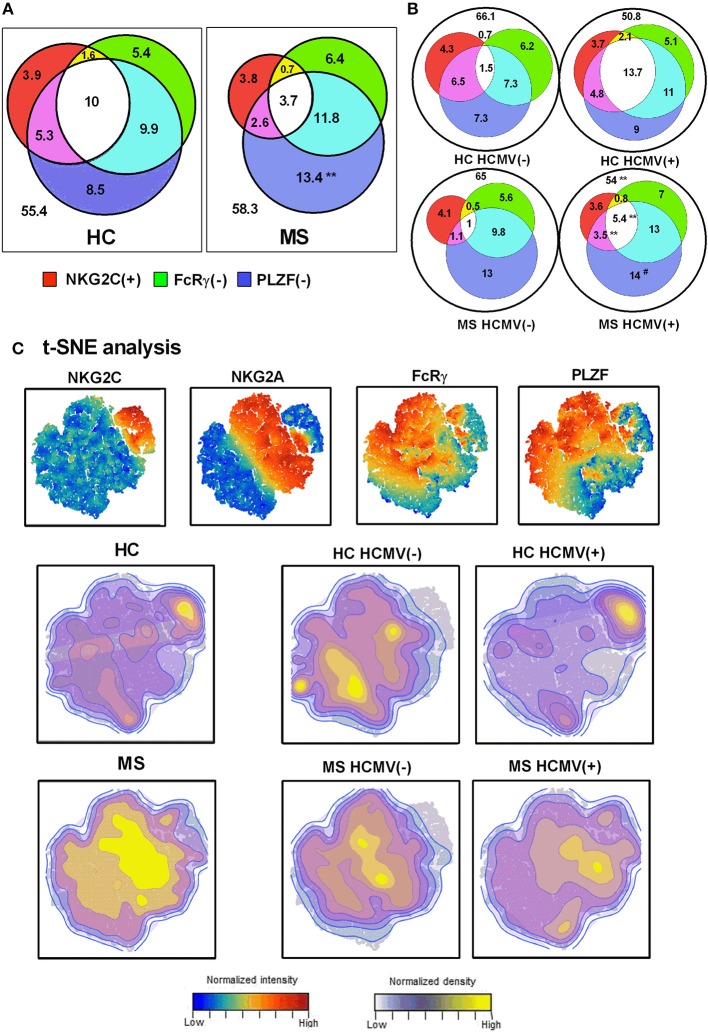
Interrelationship of adaptive NK cell markers in MS. Venn diagrams depicting the proportions of NKG2C(+), FcRγ(–), and PLZF(–) CD56^dim^ NK cells in healthy controls (HC) (*n* = 26) and MS patients (*n* = 86) **(A)**, and stratifying results according to HCMV serostatus **(B)**. Values expressed the mean proportion for each group. *P*-values (Mann–Whitney *U*-test): ** <0.01, comparing controls vs. MS patients; ## <0.01, comparing HCMV(–) vs. HCMV(+). **(C)** t-SNE plots of NKG2C, NKG2A, FcRγ, and PLZF expression in CD56^dim^ NK cells evaluated in HC and MS patients according to HCMV serostatus.

We next evaluated the relation of adaptive NK cell markers in CD56^dim^ NK cells from controls and MS patients. Three-dimensional Venn diagrams illustrated that NK cells co-expressing adaptive NK cell markers were increased in HCMV(+) compared to HCMV(–) MS patients (Figures 3A,B). The presence in MS patients of increased proportions of PLZF(–) CD56^dim^ NK cells without other adaptive markers was also confirmed (Figure 3A).

t-SNE multidimensional analysis of adaptive markers in CD56^dim^ NK cells (Figure 3C) revealed an HCMV-related expression of NKG2C, more pronounced in controls than in MS patients. By contrast, NKG2C(–) FcRγ(–) PLZF(–) NK cells were more evident in MS patients than in controls, with a less marked relation to HCMV serology (Figure 3C). These results further point out an uncoupled expression of adaptive markers in NK cells from MS patients as compared to controls, suggesting the influence of factors other than HCMV.

### Influence of IFNβ Therapy on Adaptive NK Cells in RRMS

**Figure 4:**
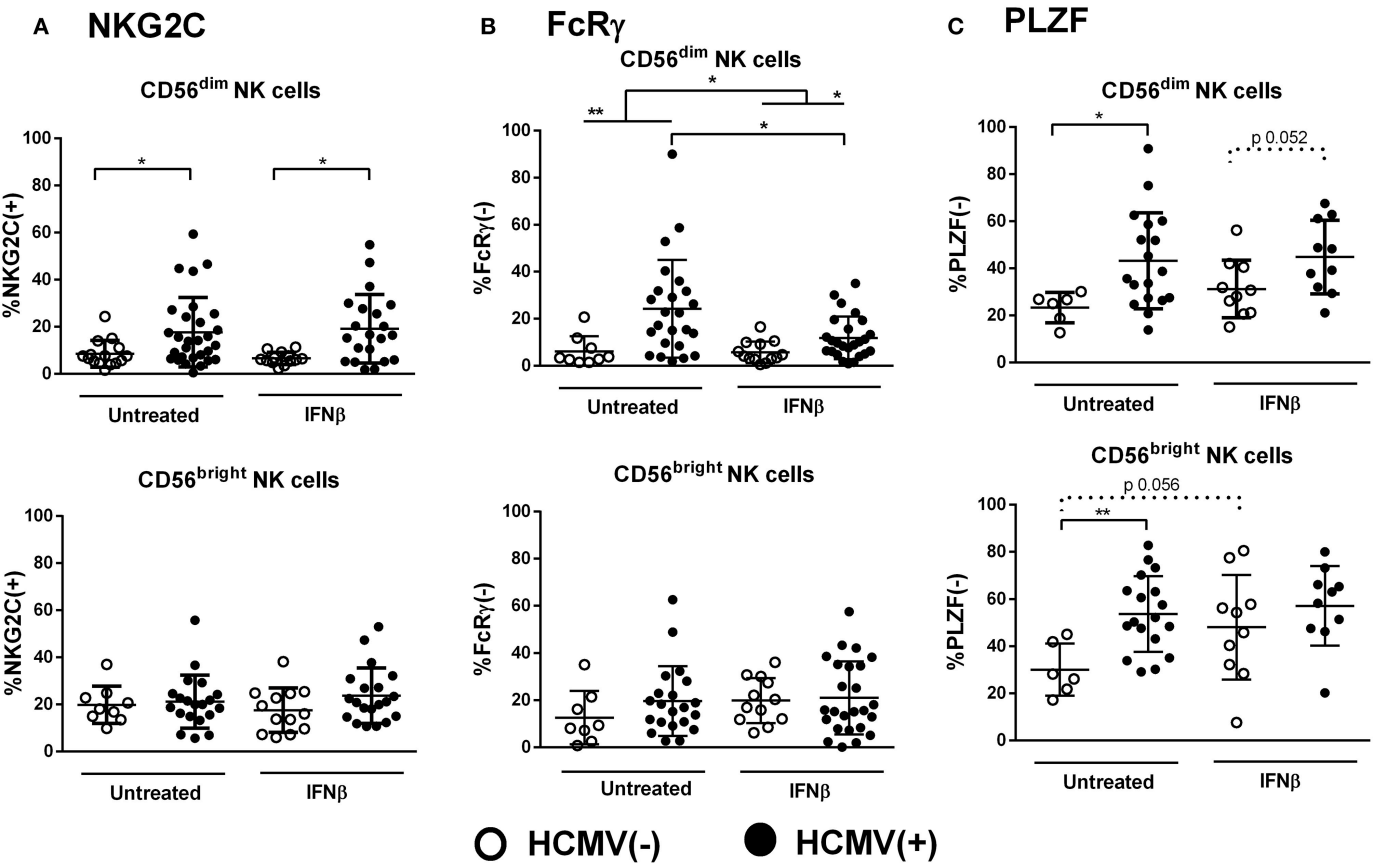
Influence of IFNβ therapy in the expression of adaptive markers in NK cells from RRMS patients. Proportions of NKG2C(+) **(A)**, FcRγ(–) **(B)**, and PLZF(–) **(C)** in CD56^dim^ and CD56^bright^ NK cells according to the presence of IFNβ therapy and HCMV serostatus. The influence of IFNβ was mainly perceived in the expression of FcRγ in CD56^dim^ NK cells in HCMV(+) cases, and in PLZF expression, which vanished differences related to HCMV serostatus in both PLZF(–) CD56^dim^ and CD56^bright^ NK cells. NKG2C(+): IFNβ-treated, *n* = 33, and untreated patients, *n* = 44; FcRγ: IFNβ-treated patients, *n* = 37, untreated patients, *n* = 33; PLZF, IFNβ-treated patients, *n* = 20, untreated patients, *n* = 24. Individuals with an *NKG2C* ^del/del^ genotype (RRMS, *n* = 5) were excluded from the analysis of NKG2C expression. *P* values (Mann–Whitney *U*-test): * <0.05, ** <0.01.

We next evaluated the impact of IFNβ therapy on adaptive NK cell markers in RRMS (mean time of treatment, 7.0 ± 4.2 years). As previously reported (36), IFNβ-treated patients displayed increased proportions of CD56^bright^ NK cells as compared to untreated cases (14.8% ± 11.6 vs. 8.3% ± 7.1, *p* < 0.05), which appeared unrelated to any of the adaptive markers analyzed (data not shown). IFNβ therapy did not alter the effect of HCMV on the proportions of NKG2C(+) CD56^dim^ NK cells (Figure 4A). However, lower proportions of FcRγ(–) CD56^dim^ NK cells were found in IFNβ-treated patients as compared to untreated cases (9.6% ± 8.2 vs. 19.3% ± 19.7, *p* < 0.05), a finding only perceived in CD56^dim^ NK cells from HCMV(+) but not in HCMV(–) MS cases, who displayed low proportions of FcRγ(–) NK cells independently of treatment (Figure 4B). Interestingly, PLZF(–) CD56^bright^ NK cells tended to increase in IFNβ-treated HCMV(–) patients (Figure 4C), vanishing the differences related to HCMV serostatus noticed in both CD56^dim^ and CD56^bright^ NK cell subsets (Figure 2C). These results suggest that HCMV-driven adaptive NK cell development in RRMS might be modulated by IFNβ therapy. However, the above-mentioned IFNβ-related modifications in adaptive markers were not perceived in a subgroup of MS patients prospectively followed after 1 year of treatment (Supplementary Figure 2), indicating that the cytokine does not directly modify the adaptive NK cell immunophenotype.

### Distribution of Adaptive NK Cells in MS Patients According to Clinical Forms

Considering MS form, NKG2C(+) CD56^dim^ NK cell proportions were lower in PPMS patients as compared to SPMS patients (Supplementary Figure 3A). By contrast, proportions of FcRγ(–) NK cells appeared greater in SPMS patients as compared to RRMS, in both CD56^dim^ and CD56^bright^ NK cells (Supplementary Figure 3B). No differences were observed for PLZF expression according to MS form (Supplementary Figure 3C). After multivariate regression analysis, the association of MS form with adaptive markers appeared independent of HCMV and IFNβ-therapy only for the proportions of NKG2C(+) CD56^dim^ and FcRγ(–) CD56^bright^ NK cells (Supplementary Table 1). No additional correlations were observed between adaptive NK cell markers and age, disease duration, disability scores, or disease activity (data not shown). These results suggest that the expression of some adaptive NK cell markers may vary in progressive MS forms independently of the influence of HCMV and IFNβ therapy.

### Adaptive NK Cell Markers and Antibody-Dependent Activation in MS

**Figure 5:**
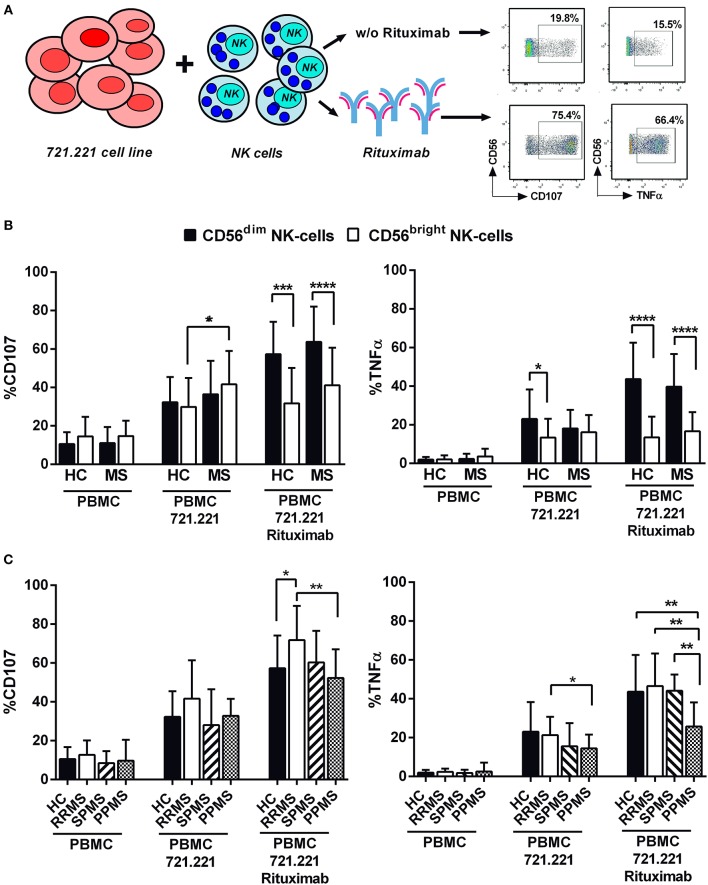
Antibody-dependent NK cell activation in MS patients and controls. **(A)** Experimental design evaluating NK cell degranulation (CD107) and intracellular TNFα production in functional experiments using 721.221 lymphoblastoid cell lines as NK cell targets. **(B)** CD107 expression and TNFα production by CD56^dim^ and CD56^bright^ NK cells in controls and MS patients. **(C)** CD107 expression and TNFα production by CD56^dim^ NK cells in controls and MS patients according to clinical form. PBMC: peripheral blood mononuclear cells. Controls, *n* = 17; MS patients, *n* = 42 (RRMS, 22; SPMS, 8, PPMS 12). *P*-values (Mann–Whitney *U*-test for CD107 and Student's *t*-test for TNFα expression): * <0.05, ** <0.01, *** <0.001, **** <0.0001.

In order to evaluate antibody-dependent NK cell functions, degranulation and TNFα production were measured in NK cells from 42 MS patients and 17 controls following stimulation with the 721.221 cell line in the absence or presence of rituximab (Figure 5A). As expected, CD56^dim^ NK cells in controls and MS patients showed a greater degranulation and TNFα production in the presence of rituximab (Figure 5B). In addition, an increased degranulation of CD56^bright^ NK cells in the direct response to the 721.221 cell line without rituximab was perceived in MS patients (Figure 5B). Comparable results were observed in experiments using the EBV-infected AKBM cell line as target (Supplementary Figure 4A).

We subsequently evaluated antibody-dependent NK cell functions according to MS form (Figure 5C). RRMS patients displayed increased proportions of degranulating CD56^dim^ NK cells as compared with controls and PPMS; moreover, the proportions of TNFα-secreting cells were lower in PPMS as compared to controls and other MS forms (Figure 5C). Similar results for TNFα production were obtained using the AKBM cell line (Supplementary Figure 4B). IFNβ therapy in MS patients did not influence the results obtained in these experiments (data not shown).

**Figure 6:**
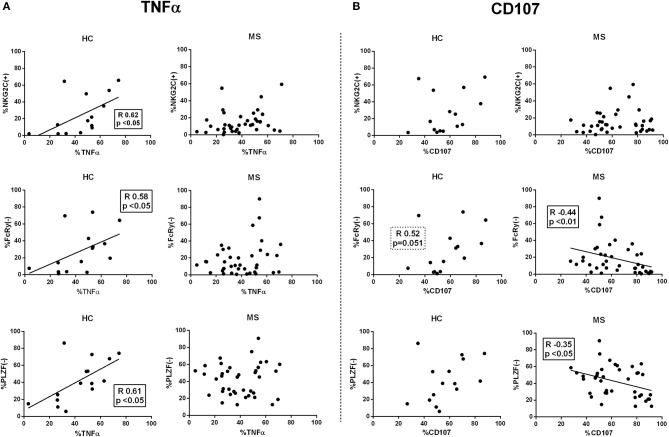
Correlations between adaptive NK markers and antibody-dependent CD56^dim^ NK cell activation. Spearman rank correlations (*R*) between proportions of NKG2C(+), FcRγ(–), and PLZF(–) CD56^dim^ NK cells and intracellular TNFα production **(A)**, and CD107 expression **(B)** in functional experiments using rituximab and 721.221 lymphoblastoid cell lines as NK cell targets. HC, healthy controls; MS, multiple sclerosis. Controls, *n* = 17; MS patients, *n* = 42 (RRMS, 22; SPMS, 8, PPMS 12).

Next, we evaluated the relationship of adaptive markers in CD56^dim^ NK cells and antibody-triggered responses. In controls, the proportions of NKG2C(+), FcRγ(–), and PLZF(–) CD56^dim^ NK cells appeared unrelated to degranulation, whereas a direct correlation with TNFα production was observed (Figure 6). By contrast, the proportions of adaptive NK cells in MS patients did not correlate with TNFα secretion and, moreover, an inverse relation between degranulation and the proportions of FcRγ(–) and PLZF(–) CD56^dim^ NK cells was detected (Figure 6); similar observations were found in CD56^bright^ NK cells from MS patients (data not shown). An analysis considering the co-expression of adaptive markers in controls and MS patients showed that antibody-triggered responses were greater in NKG2C(+) CD56^dim^ NK cells independently of FcRγ or PLZF expression (Supplementary Figure 5).

Overall, these results suggest that adaptive NK cell function may differ in MS according to clinical form, being greater in RRMS as compared to progressive MS forms; however, in contrast to healthy individuals, an adaptive NK cell profile in MS patients did not correlate with proficient antibody-triggered effector functions.

## Discussion

Considering the high prevalence of lifelong HCMV infection and its broad impact on the immune system (8), the possibility that this herpesvirus might somehow impact the development of autoimmune disorders is plausible (37). A lower HCMV seroprevalence has been reported in studies of pediatric and adult MS populations (3–7, 11), suggesting that this infection might have a protective effect. Among different hypotheses to explain how HCMV might influence MS, we considered the involvement of NK cells, which have been previously related with the disease (26–30). The virus promotes to a variable extent an adaptive differentiation of NK cells (13–15, 17, 19), which effectively mediate antibody-triggered cytotoxicity and cytokine production, thus potentially contributing to the immune response against HCMV and other microbial pathogens (10, 38–40). In line with reports relating HCMV with a reduced MS susceptibility (3–5), an association of HCMV-driven adaptive NKG2C(+) NK cell expansion with a lower risk of clinical progression was previously described (31). Yet, it remains uncertain whether and how adaptive NK cells might influence the development and clinical course of MS.

In agreement with extensive observations in healthy blood donors and different clinical settings, our results pointed out that HCMV infection is the leading factor that promotes the development of conventional adaptive NKG2C(+), FcRγ(–), and PLZF(–) CD56^dim^ NK cells in MS patients (17–19, 22, 23). In previous reports, an uncoupled distribution of the expression of some adaptive NK cell-associated markers, with potential functional implications, was noticed (17, 18, 25). In this regard, we observed increased proportions of PLZF(–) NK cells, but not of NKG2C(+) or FcRγ(–) NK cells, among the CD56^bright^ NK cell subset from HCMV(+) individuals. Such phenotype may result from an influence of HCMV at early stages of adaptive NK cell development, according to the linear model of differentiation from CD56^bright^ NK cells (41), but also of HCMV-unrelated factors (20, 42), as suggested by the detection of increased PLZF(–) CD56^dim^ NK cells in HCMV(–) MS patients.

No significant differences in NKG2C expression were perceived between controls and progressive forms; yet, lower proportions of NKG2C(+) NK cells in PPMS as compared to SPMS were detected, in line with the lower NKG2C pattern of expression in MS patients observed in the multidimensional analysis as well as in previous studies with a larger number of cases (31). An additional factor related with changes in the expression of adaptive NK cell markers in MS was IFNβ therapy, which was associated with reduced proportions of CD56^dim^ FcRγ(–) NK cells in HCMV(+) RRMS patients. Moreover, HCMV-related differences in the proportions of PLZF(–) CD56^dim^ and CD56^bright^ NK cell subsets vanished in IFNβ-treated cases, an effect related with an increase of PLZF(–) cells in HCMV(–) patients. However, no phenotypic changes in adaptive NK cells were perceived in a subset of RRMS cases prospectively followed up for 1 year after initiating IFNβ therapy, pointing out that this effect likely results from complex mechanisms eventually requiring a longer exposure to be perceived. Further studies would be required to assess whether PLZF downregulation might be related to clinical response to IFNβ therapy.

Adaptive NK cells have been reported to effectively mediate antibody-triggered effector functions, particularly cytokine production (22, 23, 34). NK cell activation upon incubation with rituximab-coated 721.221 cells was mainly exerted by CD56^dim^ NK cells, without significant differences between controls and MS patients, as previously reported (43). Other clinical variables (i.e., age and IFNβ-therapy) appeared as well unrelated with antibody-triggered responses mediated by CD56^dim^ NK cells (data not shown). Notwithstanding, NK cell activation differed in MS according to the clinical form, being lower in PPMS as compared to RRMS patients. Similar results were obtained using as a target the AKBM cell line, suggesting a comparable pattern of NK cell response against EBV-infected targets. In accordance to previous studies addressing the relation of adaptive markers with NK cell antibody-triggered function (18, 25, 44), a proficient antibody-dependent activity was related to NKG2C expression in CD56^dim^ NK cells rather than to other adaptive-associated markers. This finding, in conjunction with the low NKG2C expression found in progressive MS (31), deserves attention, suggesting that an adaptive NK cell dysfunction may be associated with a risk of disability progression.

Based on our observations, some considerations need to be made regarding the putative connection of HCMV infection and adaptive NK cells with MS immunopathology. First, the ability of NK cells to eliminate activated T lymphocytes suggested that they may play a relevant immunoregulatory role in the context of MS (27, 28, 30). Yet, adaptive NK cells do not effectively exert this function (17), possibly reflecting their expression of KIRs and ILT2 HLA class I-specific inhibitory receptors. Second, given their CD16-triggered antibody-dependent effector functions and proliferation, adaptive NK cells likely participate in the immune response to different microbial pathogens (17, 19, 22, 40). Thus, HCMV-induced adaptive NK cells might contribute to the control of other herpesviruses proposed to be involved in MS, as supported by their participation in the marked antibody-dependent response against EBV-infected cells (33). In this regard, the adaptive NK cell phenotype in MS patients was not associated with proficient antibody-dependent effector functions, in contrast to the pattern of response observed in healthy donors. Third, PLZF(–) CD56^bright^ NK cells were detected in HCMV(+) controls and MS patients and, moreover, IFNβ therapy was associated with increased PLZF(–) CD56^bright^ NK cells in HCMV(–) RRMS. This finding deserves further attention in the context of the regulatory role of CD56^bright^ NK cells and their contribution to control EBV infection (45, 46).

In conclusion, our results provide novel insights into the putative influence of HCMV in MS involving the NK cell compartment. The study shows that the distribution of adaptive NK cell markers in MS may vary not only depending on HCMV seropositivity but also on IFNβ therapy and disease form, revealing differences in their antibody-dependent activation as compared to healthy individuals. Further studies are required to explore the involvement of HCMV in the immune response to EBV in MS patients, and the putative influence of HCMV-unrelated factors in PLZF downregulation.

## Data Availability Statement

The datasets generated for this study will not be made publicly available. The work is based on flow cytometry analysis of peripheral blood mononuclear cells. Data will be available under request.

## Ethics Statement

The studies involving human participants were reviewed and approved by Comité Etic d'Investigacions Cliniques (CEIC), Hospital del Mar Medical Research Institute. The patients/participants provided their written informed consent to participate in this study.

## Author Contributions

AM, EA-P, AV, and JM-R designed the study, executed experiments, and performed the statistical analysis. AM, EA-P, ML-B, and JM-R contributed to interpretation of the results and wrote the final draft that was revised for all authors. AZ, EM, and ML contributed to data interpretation and critically reviewed the manuscript. MC-G performed and contributed to the interpretation of the t-SNE analysis. NV, LV, and RÁ-L provided samples, performed serological analysis, and critically reviewed the manuscript.

### Conflict of Interest

AM has received travel funding from Teva, Biogen Idec, Novartis, Almirall, Bayer, and Genzyme. EM has received personal fees for consulting services and lectures from Merck-Serono, Biogen Idec, Teva, Genzyme, Novartis, Bayer, and Almirall. AZ has received travel funding from Biogen Idec, Novartis, and Genzyme. RÁ-L has received honoraria for lectures from Merck-Serono, Novartis, and Biogen Idec, and grants from Merck-Serono, Teva, Sanofi-Avertis, Biogen Idec, and Bayer. LV reports personal fees for consulting services from Biogen Idec, and grants from Merck-Serono, Biogen Idec, Teva, and Genzyme. JM-R has participated as principal investigator in pharmaceutical company-sponsored clinical trials including Novartis, Roche, Merck-Serono, Actelion, and Celgene, and personal fees for consulting services and lectures from Novartis, Biogen Idec and Merck-Serono. The remaining authors declare that the research was conducted in the absence of any commercial or financial relationships that could be construed as a potential conflict of interest.
