# Supplementary material for: Adaptive Features of Natural Killer Cells in Multiple Sclerosis
Source: Front Immunol. 2019 Oct 15;10:2403. doi: 10.3389/fimmu.2019.02403 (PMC6803486; doi:10.3389/fimmu.2019.02403)
Supplement: Supplementary file 1 [file Table_1.DOCX]

**Supplementary Table 1.** **Multivariate analysis of adaptive NK cell markers in MS.**

|  | **NKG2C(+) CD56^dim^ *** | | | |  | |  | **FcRγ(-) CD56^dim^  **** | | | |  | **FcRγ(-) CD56^bright^ **** | | | | |  |  |
| --- | --- | --- | --- | --- | --- | --- | --- | --- | --- | --- | --- | --- | --- | --- | --- | --- | --- | --- | --- |
| **Univariate analysis** | **P-value †** | |  | | |  |  | **P-value †** |  | |  |  | **P-value †** | |  | |  | |  |
| **MS form** | **0.025^&^** |  |  | | | |  | **0.023^&^** |  | |  |  | **0.048^&^** |  | | |  | |  |
| **HCMV** | **<0.0001^&^** |  |  | | | |  | **<0.0001^&^** |  | |  |  | 0.263 |  | | |  | |  |
| **IFNβ** | 0.432 |  |  | | | |  | **0.010^&^** |  | |  |  | 0.746 |  | | |  | |  |
|  |  |  | |  | | |  |  |  |  | |  |  |  | |  | | |  |
| **Multivariate analysis** | **B** | **β** | | **CI 95%** | | | **p-value** | **B** | **β** | **CI 95%** | | **p-value** | **B** | **β** | | **CI 95%** | | | **p-value** |
| **MS form** | -0.219 | -0.263 | | -0.426, -0.011 | | | **0.039** | -0.067 | -0.112 | -0.175, 0.041 | | 0.221 | - | - | | - | | | **-** |
| **HCMV** | 0.105 | 0.126 | | -0.103, 0.312 | | | 0.316 | 0.448 | 0.429 | 0.273, 0.624 | | **<0.001** | - | - | | - | | | - |
| **IFNβ** | - | - | | - | | | - | -0.240 | -0.246 | -0.415, -0.065 | | **0.008** | - | - | | - | | | - |

†: Mann-Whitney test. &: variables introduced in the multivariate linear regression models. * Multivariate linear regression analysis performed in SPMS and PPMS patients (*), and in RRMS and SPMS patients (**). B: Unstandardized coefficient. β: standardized coefficient. CI 95%: confidence interval 95%.
